# No Evidence of SARS-CoV-2 Infection in Wild Mink (*Mustela lutreola* and *Neogale vison*) from Northern Spain during the First Two Years of Pandemic

**DOI:** 10.3390/ani12151971

**Published:** 2022-08-03

**Authors:** Sergio Villanueva-Saz, Jacobo Giner, Ana María Palomar, María Asunción Gómez, Madis Põdra, María del Carmen Aranda, María de los Ángeles Jiménez, Patricia Lizarraga, Raquel Hernández, Aránzazu Portillo, José Antonio Oteo, Ignacio Ruíz-Arrondo, María Dolores Pérez, Ana Pilar Tobajas, Maite Verde, Delia Lacasta, Diana Marteles, Ramón Hurtado-Guerrero, Llipsy Santiago, Héctor Ruíz, Antonio Fernández

**Affiliations:** 1 Department of Animal Pathology, Veterinary Faculty, University of Zaragoza, 50013 Zaragoza, Spain; jacoboginer@gmail.com (J.G.); mverde@unizar.es (M.V.); dlacasta@unizar.es (D.L.); diana.martelesaragues@gmail.com (D.M.); hectorruiz353@gmail.com (H.R.); afmedica@unizar.es (A.F.); 2 Clinical Immunology Laboratory, Veterinary Faculty, University of Zaragoza, 50013 Zaragoza, Spain; 3 Instituto Agroalimentario de Aragón-IA2, Universidad de Zaragoza-CITA, 50013 Zaragoza, Spain; dperez@unizar.es; 4 Infectious Diseases Department, Center of Rickettsiosis and Arthropod-Borne Diseases (CRETAV), San Pedro University Hospital-Center of Biomedical Research from La Rioja (CIBIR), Piqueras 98, 26006 Logroño, Spain; aportillo@riojasalud.es (A.P.); jaoteo@riojasalud.es (J.A.O.); irarrondo@riojasalud.es (I.R.-A.); 5 Tragsatec, Tragsatec, Division of Environmental Services, Julian Camarillo 6A-4A Planta, Sector B, 28037 Madrid, Spain; asun_emink@yahoo.es (M.A.G.); madis.podra@yahoo.es (M.P.); raquelhernandez1992@gmail.com (R.H.); 6 Fundación para la Investigación en Etología y Biodiversidad, 45950 Toledo, Spain; carmen.aranda@fiebfoundation.org; 7 Departamento Medicina y Cirugía Animal, Facultad de Veterinaria, Universidad Complutense de Madrid, Av. Puerta de Hierro s/n, 28040 Madrid, Spain; mariadji@vet.ucm.es; 8 Centro de Recuperación de Fauna de Martioda, Martioda Entitatea 3, 01191 Martioda, Spain; lizarmen79@gmail.com; 9 Department of Animal Production and Sciences of the Food, Veterinary Faculty, University of Zaragoza, 50013 Zaragoza, Spain; atobajas@unizar.es; 10 Institute for Biocomputation and Physics of Complex Systems (BIFI), Mariano Esquillor s/n, Campus Rio Ebro, Edificio I+D, University of Zaragoza, 50018 Zaragoza, Spain; rhurtado@bifi.es; 11 Aragon I+D Foundation (ARAID), 50018 Zaragoza, Spain; 12 Laboratorio de Microscopías Avanzada (LMA), University of Zaragoza, 50018 Zaragoza, Spain; 13 Copenhagen Center for Glycomics, Department of Cellular and Molecular Medicine, University of Copenhagen, 2200 Copenhagen, Denmark; 14 Aragon Health Research Institute (IIS Aragón), 50009 Zaragoza, Spain; llipsysg@gmail.com

**Keywords:** coronavirus disease 2019 (COVID-19), ELISA, mink, RT-qPCR, SARS-CoV-2, serology, Spain

## Abstract

**Simple Summary:**

The severe acute respiratory syndrome coronavirus-2 (SARS-CoV-2) causing coronavirus disease-2019 (COVID-19) is a betacoronavirus (β-CoV) closely related to Severe Acute Respiratory Syndrome (SARS-CoV) and the Middle East Respiratory Syndrome Coronavirus (MERS-CoV), which have also caused severe outbreaks of disease in human populations. Human-to-animal transmission events during the COVID-19 pandemic have been documented in several countries. Different animal species have been proven to be susceptible to infection with SARS-CoV-2 both naturally and by experimental infection, including mustelids such as ferrets, otters, and American mink (*Neogale vison*). In this sense, infected farmed American mink develop respiratory signs associated with viral pneumonia. This study evaluates the presence of SARS-CoV-2 in European mink (*Mustela lutreola*) and American mink from Spain, by enzyme-linked immunosorbent assay (ELISA) using the receptor binding domain (RBD) of Spike protein antigen in serum samples and/or by RT-qPCR assays in oropharyngeal and rectal swabs. From January 2020 to February 2022, a total of 162 animals (127 European mink and 35 American mink) with no evidence of SARS-CoV-2 infection were included in the study. Of the 126 serum samples analysed by serology, anti-SARS-CoV-2 antibodies were not detected in the mink included in this study. In the same way, SARS-CoV-2 RNA has not been detected in any of the 160 swabs samples analysed by RT-qPCR. This study shows the absence of the wild mink exposure to SARS-CoV-2 in a geographic area seriously affected by COVID-19. With these results, it can be considered that the probability that the virus is circulating in wild mink is low. With this, the risk of virus transmission to humans by this route is also considered improbable.

**Abstract:**

The impact of the SARS-CoV-2 pandemic on wildlife is largely unevaluated, and extended surveillance of animal species is needed to reach a consensus on the role of animals in the emergence and maintenance of SARS-CoV-2. This infection has been detected in farmed and domestic animals and wild animals, mainly in captivity. The interactions or shared resources with wildlife could represent a potential transmission pathway for the SARS-CoV-2 spill over to other wild species and could lead to health consequences or the establishment of new reservoirs in susceptible hosts. This study evaluated the presence of SARS-CoV-2 in European mink (*Mustela lutreola*) and American mink (*Neogale vison*) in Spain by enzyme-linked immunosorbent assay (ELISA) using the receptor binding domain (RBD) of Spike antigen in serum samples and/or by RT-qPCR assays in oropharyngeal and rectal swabs. From January 2020 to February 2022, a total of 162 animals (127 European mink and 35 American mink) with no evidence of SARS-CoV-2 infection were included in the study. Antibodies against the SARS-CoV-2 were not found in the serum samples analysed (*n* = 126), nor was the virus amplified by RT-qPCR (*n* = 160 swabs). Our results suggest that the potential role of wild mink and the European mink bred in captivity and released to the wild as dispersers of SARS-CoV-2 is so far low. However, wildlife surveillance for early detection of human and animal risks should be continued. In this sense, epidemiological monitoring measures, including serology and molecular analysis, are necessary.

## 1. Introduction

The severe acute respiratory syndrome coronavirus-2 (SARS-CoV-2) causing coronavirus disease-2019 (COVID-19) is a betacoronavirus (β-CoV) closely related to Severe Acute Respiratory Syndrome (SARS-CoV) and the Middle East Respiratory Syndrome Coronavirus (MERS-CoV), which have also caused severe outbreaks in human populations. Human-to-animal transmission events during the COVID-19 pandemic have been documented in several countries, including Hong Kong, Belgium, the United States, Netherlands, Denmark, Spain, Germany, and France, among others [1,2]. Furthermore, several animal species have proven to be susceptible to SARS-CoV-2 infection, both naturally and experimentally [2,3], including mustelids such as farmed American mink (*Neogale vison*) [4,5], mink and ferret under experimental conditions [6,7,8,9,10], household and kept ferrets [11,12], captive Asian small-clawed otters [13], wild Eurasian river otter (*Lutra lutra*) [14] and feral American mink [15,16]. In this sense, the virus spreads efficiently in farmed American mink. Furthermore, the high density of farm animals provides suitable conditions for mink-to-mink virus transmission via different routes such as droplets [1,10,17]. Furthermore, the ongoing farm-to-farm spread has been observed, and investigations are exploring the transmission routes between farms [18]. The first outbreak of SARS-CoV-2 infection in farmed mink was reported in The Netherlands in April 2020 [5]. Subsequently, it has been described in several European countries and North America [19,20,21]. Mink-to-human transmission of SARS-CoV-2 was initially documented in Denmark, where mink infected two workers on a farm, and the virus was subsequently spread in the community [18]. Genetic studies of the virus suggest the infection of mink from humans, the subsequent adaptation of the virus to the new host and the last spill over to humans [22,23].

Reports of SARS-CoV-2 infection and COVID-19 symptoms in wildlife and domestic animals across the globe is well documented [24,25,26]. Among wildlife, American mink [27] and Eurasian river otter [14] are the only free-living animals that have been reported to be infected with SARS-CoV-2 in Spain. Although there is no evidence that SARS-CoV-2 is circulating or has been established in wild animal populations, the possibility of such a scenario cannot be ruled out [27]. Therefore, this study evaluated the presence of SARS-CoV-2 infection in critically endangered European mink (*Mustela lutreola*) and feral American mink from Spain by enzyme-linked immunosorbent assay (ELISA) using the receptor binding domain (RBD) of Spike protein antigen and/or quantitative reverse transcription polymerase chain reaction (RT-qPCR).

## 2. Materials and Methods

### 2.1. Study Area and Data Collection

The study was conducted in northern Spain within the distribution area of European mink and surroundings [28]. Samples from feral American mink were collected during the trapping and culling campaign in the Basque Country (province of Alava), La Rioja and Aragon (province of Teruel). In the case of the European mink, samples were taken from wild and captive-bred individuals. The latter belonged to the National European mink Breeding Program (MARM 2008) and included crossbred individuals between Spanish and EEP (European Endangered Species Program) populations [29]. Wild European mink were live-trapped during periodic surveys carried out in the Basque Country (province of Alava), La Rioja, Navarre and Aragon (province of Zaragoza). A few non-target individuals were captured during the American mink trapping campaigns too. Furthermore, samples from captive-born individuals were taken before their release (a conservation initiative that aims to improve the status of the species) in the Basque Country (province of Alava), La Rioja and Aragon (crossbred mink were released in the provinces of Zaragoza and Huesca). In addition, all the individuals maintained in the breeding centre of Foundation for Research in Ethology and Biodiversity (FIEB) located in Casarrubios del Monte (Toledo, Spain) were sampled.

Samples from European and American mink screened for SARS-CoV-2 were collected from January 2020 to February 2022. Data such as species identification and geographic origin were recorded for every animal. This survey was performed during the regular processes carried out in the programmes for the conservation of the European mink and the control of the American mink. These programmes were authorized and supported by the Spanish Government (18MNES002; Ministry for the Ecological Transition and the Demographic Challenge), and Regional-Governments (09-7-4-02-0051/2020, 09-7-4.02-0034/2021, A/2021/030, A/2022/030; Diputación Foral de Álava, Gobierno de La Rioja, Gobierno de Aragón and Gobierno de Navarra). The care and use of animals were performed according to the Spanish Policy for Animal Protection RD 53/2013, which meets the European Union Directive 2010/63 on the protection of animals used for experimental and other scientific purposes.

### 2.2. Sampling

Serum samples were collected from mink and tested with an in-house ELISA for anti-SARS-CoV-2 antibodies detection in the Department of Animal Pathology of the University of Zaragoza. Moreover, oropharyngeal and rectal swabs were obtained to perform RT-qPCR specific for SARS-CoV-2 in the Center of Rickettsiosis and Arthropod-Borne Diseases Center of Biomedical Research of La Rioja (CRETAV-CIBIR). Finally, it is worth mentioning that all the specimens from the National European mink Breeding Programme released during this period were confirmed to be negative for SARS-CoV-2 infection by RT-qPCR assays before their liberation.

Wild mink, including both species, were captured in 15 × 15 × 60 cm single entry live traps, as described previously [30]. All European mink were clinically examined, bled by jugular puncture, and marked with subcutaneous passive transponder tags for identification. Captive-bred mink samples were obtained during routine healthcare check-ups of mink from the FIEB and during the check-up and tagging of the individuals destined to release operations. Samplings (blood and swabs) were performed under inhalation anaesthesia with isoflurane. Blood samples were collected aseptically by cranial cava venepuncture to perform routine laboratory tests such as a complete blood cell count and biochemical profile. After recovery from anaesthesia, they were released at their capture locations. American mink were euthanized, and blood samples were collected from cardiac puncture and were stored at −20 °C until tested.

Moreover, swabs samples were obtained. Those that were processed or frozen in less than 5 h were preserved in Dulbecco’s modified Eagle medium (DMEM) with penicillin (100 units/mL) and streptomycin (100 μg/mL) (Sigma-Aldrich, St. Louis, MO, USA) [26]. In specific cases in which the quick processing of the sample could not be guaranteed, swabs with transport medium (∑-Virocult^®^ 951S, MWE Medical Wire, Corsham, UK) were used and preserved at 4 °C before the analysis. After data collection and under anaesthesia, animals were euthanized following the welfare legal standards. The study was conducted according to the guidelines of the Declaration of Helsinki. 

### 2.3. Expression and Purification of Receptor Binding Domain (RBD) of Spike Protein

The DNA sequence encoding amino acid residues 319-541 (RVQPTESIVRFPNITNLCPFGEVFNATRFASVYAWNRKRISNCVADYSVLYNSASFSTFKCYGVSPTKLNDLCFTNVYADSFVIRGDEVRQIAPGQTGKIADYNYKLPDDFTGCVIAWNSNNLDSKVGGNYNYLYRLFRKSNLKPFERDISTEIYQAGSTPCNGVEGFNCYFPLQSYGFQPTNGVGYQPYRVVVLSFELLHAPATVCGPKKSTNLVKNKCVNF) of the RBD was codon optimized and synthesized by Gen-Script (Piscataway, NJ, USA) for expression in HEK293 cells, as described previously [11,31,32].

### 2.4. Detection of SARS-CoV-2 Antibodies by in-House ELISA

An in-house indirect ELISA for the detection of IgG specific to RBD of Spike protein was established with some modifications [11,31,32]. As a positive control, each plate included two serum samples, including a human patient diagnosed with COVID-19, confirmed by a molecular test and a commercial quantitative ELISA, and two serum samples from a seropositive cat [31] and a seropositive ferret to SARS-CoV-2 [11]. The same positive and negative sera were used for all assays. All samples were run in duplicate. The cut-off for European mink was set to 0.193 Optical Density units (OD units) (mean +3 standard deviations of values from 27 European mink obtained before the COVID-19 situation in 2020). By contrast, the cut-off for American mink was set to 0.150 Optical Density units (OD units) (mean +3 standard deviations of values from 47 American mink obtained prior the COVID-19 situation in 2020). In both cases, the results above this value were considered positive.

### 2.5. SARS-CoV-2 RT-qPCR Assays

Oropharyngeal and rectal swabs were extracted as described in a previous work [26]. Samples were screened for SARS-CoV-2 RNA detection using two specific one-step RT-qPCR assays targeting gene fragments encoding the nucleocapsid (N) and the envelope (E) protein-encoding as previously described [33]. Two different target assays were used to reduce the possibility of false-negative PCR results. These two popular RT-qPCR assays were selected according to our experience and their adequate specificity, efficiency, and sensitivity [34]. Moreover, the amplification of the β-actin mRNA was used as internal control [35]. Positive [synthetic plasmid controls with the complete SARS-CoV-2 N gene (Integrated DNA Technologies, Leuven, Belgium) and the E gene (Eurofins Genomics Germany GmbH)] and negative (extraction and amplification) controls were included in the assays. Samples and controls were tested in triplicate.

## 3. Results

### 3.1. Characterization of the Analysed Animals

A total of 162 mink (127 European mink and 35 American mink) were included in this study. All the American mink and 57 European mink were live-trapped in the wild. The remaining 70 European mink analysed belonged to the breeding programme, 53 of them were studied before their release (Table 1). All tested mink were assessed as apparently healthy, with no evident systemic signs found during the general physical examination.

### 3.2. Serological and Molecular Prevalence of SARS-CoV-2 Infection

Of the 126 serum samples (101 European mink and 25 American mink) analysed by serology, anti-SARS-CoV-2 antibodies were not detected in the mink included in this study. Similarly, SARS-CoV-2 RNA was not detected in any of the 160 swabs samples (59 European mink and 21 American mink) analysed by RT-qPCR. The β-actin was amplified in all the samples. The mink individuals analysed with the different techniques used in this study are detailed in Table 1.

## 4. Discussion

This is one of the few studies assessing SARS-CoV-2 infection including serological and molecular analysis in a large wild mink population. American mink are highly susceptible to SARS-CoV-2, and this virus spreads efficiently within mink farms once introduced by direct and indirect contact [36]. The SARS-CoV-2 virus has been detected in more than 400 mink farms in eight countries in the European Union, including Spain and North America [19,20,37], and reinfections have been demonstrated [38]. The transfer of variants between humans and mink has been reported in Denmark, The Netherlands and Poland [18,23,39,40]. Moreover, the infection of feral cats from mink on a farm has been also suggested [41]. Clinical signs of infected farmed American mink may range from absent to mild, moderate, and even fatal, with a mortality rate from 1.2% to 2.4% [3]. However, symptomatic infected mink could exhibit mainly respiratory or gastrointestinal signs [20,26,42,43,44].

Wild mustelid species, widely distributed in Europe, might approach mink farms and eventually acquire SARS-CoV-2 from infected farmed animals, farm-escaped free-ranging American mink or animal products. Thus, an asymptomatic infection was confirmed in an American mink by real time RT-PCR and sequencing using nasal swab sampled in Utah as part of wildlife surveillance around infected mink farms [15]. In eastern Spain, the virus was amplified from mesenteric lymph nodes of 2 out of 13 feral American mink using a two-step manual RT-PCR assay. However, two commercial one-step RT-PCR assays gave negative results [16]. Moreover, the virus was detected in an adult male Eurasian river otter freshly road-killed in the same area [14].

Similarly, SARS-CoV-2 infection has been confirmed in household domestic ferrets [11] and in ferrets kept for hunting purposes in Spain [12]. Those animals used for hunting have close contact with their human keepers with the potential for escaping into the wild and interacting with wild mammals. Nevertheless, due to their elusive and solitary behaviour and low density, there is likely low risk of contact with humans and/or other SARS-CoV-2 susceptible hosts. Therefore, the risk of wild mustelids becoming a reservoir for SARS-CoV-2 in Europe is low [36].

Equally, there are far fewer opportunities for transmission from humans to free-living wildlife, but some activities involving direct contact could suppose significant risks. Indirect transmission might also occur where there are opportunities for human contamination of the environment, food transmission, urban waste, or fomites [45].

For these reasons, the transmission of SARS-CoV-2 from humans, farmed or kept animals to wild animals should be monitored. SARS-CoV-2 early detection in wild mink (feral American mink and European mink) aims to find the virus infection as soon as possible after it enters the wild to safeguard animal and public health.

In Spain, the number of mink farms with a high density of animals, the susceptibility of mink to the SARS-CoV-2 virus infection, together with the high number of human cases of COVID-19 reported should be evaluated. Furthermore, the proven bidirectional transmission of the virus between animals and humans suggests an increased risk of SARS-CoV-2 in farms within mink, humans, and domestic animals. Although the risk of the virus dispersion from farms to wildlife decreases [46,47,48] but it is not null, there remains a risk of virus reservoir establishment in feral mink.

The European mink is one of the most threatened mammal species in Europe, listed as critically endangered by the European Commission and carried out by the World Conservation Union (IUCN), with a fragmented distribution in a few European countries [49]. The species is negatively affected mostly by direct aggression from feral American mink. Spain holds one of the last populations of European mink in areas where American mink is expanding [28,49]. Conservation interventions on the species in this country include the breeding programme with translocation attempts and eradicating the American mink populations. Although the alien mink is the main threat, some other risks such as like habitat loss [50] may have a significant negative impact on the remaining populations of the European mink due to its critical conservation status. Cases of SARS-CoV-2 infections could also affect this endangered species in the wild and conservation breeding facilities. Therefore, prevention and control of the virus circulation in wild populations is, at the moment, significant to preserving this species. In addition, during this pandemic, the Spanish Programmes for the conservation of the European mink and the control of the American mink have established protocols to prevent the SARS-CoV-2 transmission in the few processes that require any human contact with these animals. These measures include the screening of SARS-CoV-2 in captive and feral mink. In this study, using a specific SARS-CoV-2 ELISA, none of the tested mink displayed anti-SARS-CoV-2 serum antibodies. Equally, RT-qPCR assays performed on European and American mink in this study resulted negative, including captive individuals. The combination of molecular and serological tests in different types of samples is an optimal diagnostic approach to maximize the possibility of virus detection. Our data suggest that the role of wild mink in the epidemiology of the SARS-CoV-2 virus is low. Thus, the captive-breeding of the European mink carried out in the studied area requires strictly regulated contact with staff to avoid the possible virus spread. Still, the negative results of the present study demonstrate the efficiency of the established protocols for preventing virus transmission among animals or the bidirectional transmission between humans and mink.

## 5. Conclusions

This study shows the absence of exposure to SARS-CoV-2 in wild mink in a geographic area seriously affected by COVID-19. With these results, it can be considered that so far, the probability that the virus is circulating in wild mink is very low. Thus, the risk of transmission of the virus to people by this route is also considered improbable since the contact between mink and humans in nature is, in practice, non-existent. In this sense, the application of measures to prevent the transmission of SARS-CoV-2 from personnel handling animals during the release process seems appropriate. However, further studies are necessary to evaluate the impact of SARS-CoV-2 in these and other wild mammal species. Moreover, when humans have close contact with wildlife, the risk of spreading SARS-CoV-2 to animals, and the potential spillback to people, increases. Therefore, wildlife authorities should consider using appropriate personal protective equipment when humans are going to be exposed to direct contact with wildlife animals.

## Figures and Tables

**Table 1:** Number of mink analysed in this study using different techniques for the SARS-CoV-2 antibody and nucleic acid detection.

Species	Origin	Year	Province (Autonomous Community)	Techniques		
				ELISA	RT-qPCR	ELISA & RT-qPCR
European mink	Released	2020	Alava (BC)	3	0	0
		2020	La Rioja	0	6	4
		2021	Alava (BC)	0	7	1
		2021	La Rioja (LR)	0	7	2 ^†^
		2021	Zaragoza and Huesca (Aragón)	1	6	16
	Total			4	26	23
	Captured	2020	Alava (BC)	11	0	0
		2020	La Rioja (LR)	18 ^‡^	0	0
		2020	Navarre (N)	5	0	0
		2020	Zaragoza(A)	3	0	0
		2021	Alava (BC)	1	0	0
		2021	La Rioja (LR)	9	4 ^‡,§^	5
		2021	Navarre (N)	2	0	0
		2021	Zaragoza (A)	1	0	0
	Total			48	2 ^§^	7 ^‡^
	Captive	2020		2 ^¶^	0	0
		2021		16 ^¶^	0	0
	Total			17 ^¶^	0	0
	Total European mink			69 ^¶^	28 ^§^	30 ^†,‡^
American mink	Captured	2020	Alava (BC)	1	0	0
		2020	La Rioja (LR)	0	7	0
		2021	La Rioja (LR)	0	0	11
		2021	Teruel (A)	13	0	0
		2022	La Rioja (LR)	0	3	0
	Total American mink			14	10	11
Total				83 ^¶^	38 ^§^	41 ^†,‡^

ELISA: enzyme-linked immunosorbent assay; RT-qPCR: quantitative reverse transcription polymerase chain reaction; BC: Basque Country; LR: La Rioja; A: Aragon; N: Navarre; ^†^ One specimen was tested twice by ELISA (March and September 2021), but only once by RT-qPCR (September 2021); ^‡^ Two specimens were tested by ELISA in 2020 and by RT-qPCR in 2021. They have been counted only in the total number of specimens analysed by ELISA & RT-qPCR; ^§^ One specimen was tested twice (April and September 2021); ^¶^ One specimen was tested in two consecutive years. It has been counted only once in the total number.

## Data Availability

The data presented in this study are available on request from the corresponding author. The data are not publicly available due to conservation wildlife programmes.

## References

[1] Jo W.K., de Oliveira-Filho E.F., Rasche A., Greenwood A.D., Osterrieder K., Drexler J.F. (2021). Potential zoonotic sources of SARS-CoV-2 infections. Transbound. Emerg. Dis..

[2] Mastutik G., Rohman A., I’tishom R., Ruiz-Arrondo I., de Blas I. (2022). Experimental and natural infections of severe acute respiratory syndrome-related coronavirus 2 in pets and wild and farm animals. Vet. World..

[3] Islam A., Ferdous J., Islam S., Sayeed M.A., Rahman M.K., Saha O., Hassan M.M., Shirin T. (2021). Transmission dynamics and susceptibility patterns of SARS-CoV-2 in domestic, farmed and wild animals: Sustainable One Health surveillance for conservation and public health to prevent future epidemics and pandemics. Transbound. Emerg. Dis..

[4] Molenaar R.J., Vreman S., Hakze-van der Honing R.W., Zwart R., de Rond J., Weesendorp E., Smit L., Koopmans M., Bouwstra R., Stegeman A. (2020). Clinical and Pathological Findings in SARS-CoV-2 Disease Outbreaks in Farmed Mink (*Neovison vison*). Vet. Pathol..

[5] Oreshkova N., Molenaar R.J., Vreman S., Harders F., Oude Munnink B.B., Hakze-van der Honing R.W., Gerhards N., Tolsma P., Bouwstra R., Sikkema R.S. (2020). SARS-CoV-2 infection in farmed minks, the Netherlands, April and May 2020. Eurosurveillance.

[6] Kim Y.I., Kim S.G., Kim S.M., Kim E.H., Park S.J., Yu K.M., Chang J.H., Kim E.J., Lee S., Casel M. (2020). Infection and Rapid Transmission of SARS-CoV-2 in Ferrets. Cell Host Microbe.

[7] Richard M., Kok A., de Meulder D., Bestebroer T.M., Lamers M.M., Okba N., Fentener van Vlissingen M., Rockx B., Haagmans B.L., Koopmans M. (2020). SARS-CoV-2 is transmitted via contact and via the air between ferrets. Nat. Commun..

[8] Sarkar J., Guha R. (2020). Infectivity, virulence, pathogenicity, host-pathogen interactions of SARS and SARS-CoV-2 in experimental animals: A systematic review. Vet. Res. Commun..

[9] Shi J., Wen Z., Zhong G., Yang H., Wang C., Huang B., Liu R., He X., Shuai L., Sun Z. (2020). Susceptibility of ferrets, cats, dogs, and other domesticated animals to SARS-coronavirus 2. Science.

[10] Shuai L., Zhong G., Yuan Q., Wen Z., Wang C., He X., Liu R., Wang J., Zhao Q., Liu Y. (2020). Replication, pathogenicity, and transmission of SARS-CoV-2 in minks. Natl. Sci. Rev..

[11] Giner J., Villanueva-Saz S., Tobajas A.P., Pérez M.D., González A., Verde M., Yzuel A., García-García A., Taleb V., Lira-Navarrete E. (2021). SARS-CoV-2 Seroprevalence in Household Domestic Ferrets (*Mustela putorius furo*). Animals.

[12] Gortázar C., Barroso-Arévalo S., Ferreras-Colino E., Isla J., de la Fuente G., Rivera B., Domínguez L., de la Fuente J., Sánchez-Vizcaíno J.M. (2021). Natural SARS-CoV-2 infection in kept ferrets, Spain. Emerg. Infect. Dis..

[13] USDA-APHIS Animal and Plant Health Inspection Service U.S. Department of Agriculture. https://www.aphis.usda.gov/aphis/newsroom/stakeholder-info/SA_By_Date/SA-2021/SA-04.

[14] Padilla-Blanco M., Aguiló-Gisbert J., Rubio V., Lizana V., Chillida-Martínez E., Cardells J., Maiques E., Rubio-Guerrí C. (2022). The Finding of the Severe Acute Respiratory Syndrome Coronavirus (SARS-CoV-2) in a Wild Eurasian River Otter (*Lutra lutra*) Highlights the Need for Viral Surveillance in Wild Mustelids. Front. Vet. Sci..

[15] Shriner S.A., Ellis J.W., Root W., Stopak S.R., Wiscomb G.W., DeLiberto T.J. (2021). SARS-CoV-2 exposure in escaped mink, Utah, USA. Emerg. Infect. Dis..

[16] Aguiló-Gisbert J., Padilla-Blanco M., Lizana V., Maiques E., Muñoz-Baquero M., Chillida-Martínez E., Cardells J., Rubio-Guerri C. (2021). First Description of SARS-CoV-2 Infection in two feral American Mink (*Neovison vison*) caught in the wild. Animals.

[17] Enserink M. (2020). Coronavirus rips through Dutch mink farms, triggering culls. Science.

[18] Oude Munnink B.B., Sikkema R.S., Nieuwenhuijse D.F., Molenaar R.J., Munger E., Molenkamp R., van der Spek A., Tolsma P., Rietveld A., Brouwer M. (2021). Transmission of SARS-CoV-2 on mink farms between humans and mink and back to humans. Science.

[19] Pomorska-Mól M., Włodarek J., Gogulski M., Rybska M. (2021). Review: SARS-CoV-2 infection in farmed minks-an overview of current knowledge on occurrence, disease and epidemiology. Animals.

[20] Fenollar F., Mediannikov O., Maurin M., Devaux C., Colson P., Levasseur A., Fournier P.E., Raoult D. (2021). Mink, SARS-CoV-2, and the Human-Animal Interface. Front. Microbiol..

[21] ECCD (European Centre for Disease Prevention and Control, Stockholm 2020. https://www.ecdc.europa.eu/sites/default/files/documents/RRA-SARS-CoV-2-in-mink-12-nov-2020.pdf.

[22] Burkholz S., Pokhrel S., Kraemer B.R., Mochly-Rosen D., Carback R.T., Hodge T., Harris P., Ciotlos S., Wang L., Herst C.V. (2021). Paired SARS-CoV-2 spike protein mutations observed during ongoing SARS-CoV-2 viral transfer from humans to minks and back to humans. Infect. Genet. Evol..

[23] Hammer A.S., Quaade M.L., Rasmussen T.B., Fonager J., Rasmussen M., Mundbjerg K., Lohse L., Strandbygaard B., Jørgensen C.S., Alfaro-Núñez A. (2021). SARS-CoV-2 Transmission between Mink (*Neovison vison*) and Humans, Denmark. Emerg. Infect. Dis..

[24] Kumar A., Pandey S.N., Pareek V., Narayan R.K., Faiq M.A., Kumari C. (2021). Predicting susceptibility for SARS-CoV-2 infection in domestic and wildlife animals using ACE2 protein sequence homology. Zoo Biol..

[25] Tiwari R., Dhama K., Sharun K., Iqbal Yatoo M., Malik Y.S., Singh R., Michalak I., Sah R., Bonilla-Aldana D.K., Rodriguez-Morales A.J. (2020). COVID-19: Animals, veterinary and zoonotic links. Vet. Q..

[26] Yoo H.S., Yoo D. (2020). COVID-19 and veterinarians for one health, zoonotic- and reverse-zoonotic transmissions. J. Vet. Sci..

[27] Sharun K., Dhama K., Pawde A.M., Gortázar C., Tiwari R., Bonilla-Aldana D.K., Rodriguez-Morales A.J., de la Fuente J., Michalak I., Attia Y.A. (2021). SARS-CoV-2 in animals: Potential for unknown reservoir hosts and public health implications. Vet. Q..

[28] Põdra M., Gómez A. (2018). Rapid expansion of the American mink poses a serious threat to the European mink in Spain. Mammalia.

[29] Tragsatec (2019). Nuevos Enfoques en la Conservación del Visón Europeo en España.

[30] Giner J., Villanueva-Saz S., Fernández A., Gómez M.A., Podra M., Lizarraga P., Lacasta D., Ruiz H., Del Carmen Aranda M., de Los Ángeles Jimenez M. (2022). Detection of anti-*Leishmania infantum* antibodies in wild European and American Mink (*Mustela lutreola* and *Neovison vison*) from Northern Spain, 2014–2020. J. Wildl. Dis..

[31] Villanueva-Saz S., Giner J., Tobajas A.P., Pérez M.D., González-Ramírez A.M., Macías-León J., González A., Verde M., Yzuel A., Hurtado-Guerrero R. (2021). Serological evidence of SARS-CoV-2 and co-infections in stray cats in Spain. Transbound. Emerg. Dis..

[32] Villanueva-Saz S., Giner J., Fernández A., Lacasta D., Ortín A., Ramos J.J., Ferrer L.M., Ruiz de Arcaute M., Tobajas A.P., Pérez M.D. (2021). Absence of SARS-CoV-2 Antibodies in Natural Environment Exposure in Sheep in Close Contact with Humans. Animals.

[33] Ruiz-Arrondo I., Portillo A., Palomar A.M., Santibáñez S., Santibáñez P., Cervera C., Oteo J.A. (2021). Detection of SARS-CoV-2 in pets living with COVID-19 owners diagnosed during the COVID-19 lockdown in Spain: A case of an asymptomatic cat with SARS-CoV-2 in Europe. Transbound. Emerg. Dis..

[34] Barra G.B., Santa Rita T.H., Mesquita P.G., Jácomo R.H., Nery L.F.A. (2020). Analytical Sensitivity and Specificity of Two RT-qPCR Protocols for SARS-CoV-2 Detection Performed in an Automated Workflow. Genes.

[35] Toussaint J.F., Sailleau C., Breard E., Zientara S., De Clercq K. (2007). Bluetongue virus detection by two real-time RT-qPCRs targeting two different genomic segments. J. Virol. Methods..

[36] Boklund A., Hammer A.S., Quaade M.L., Rasmussen T.B., Lohse L., Strandbygaard B., Jørgensen C.S., Olesen A.S., Hjerpe F.B., Petersen H.H. (2021). SARS-CoV-2 in Danish Mink Farms: Course of the Epidemic and a Descriptive Analysis of the Outbreaks in 2020. Animals.

[37] Badiola J.J., Otero A., Sevilla E., Marín B., García Martínez M., Betancor M., Sola D., Pérez Lázaro S., Lozada J., Velez C. (2022). SARS-CoV-2 Outbreak on a Spanish Mink Farm: Epidemiological, Molecular, and Pathological Studies. Front. Vet. Sci..

[38] Rasmussen T.B., Fonager J., Jørgensen C.S., Lassaunière R., Hammer A.S., Quaade M.L., Boklund A., Lohse L., Strandbygaard B., Rasmussen M. (2021). Infection, recovery and re-infection of farmed mink with SARS-CoV-2. PLoS Pathog..

[39] Koopmans M. (2021). SARS-CoV-2 and the human-animal interface: Outbreaks on mink farms. Lancet. Infect. Dis..

[40] Rabalski L., Kosinski M., Mazur-Panasiuk N., Szewczyk B., Bienkowska-Szewczyk K., Kant R., Sironen T., Pyrc K., Grzybek M. (2022). Zoonotic spill-over of SARS-CoV-2: Mink-adapted virus in humans. Clin. Microbiol. Infect..

[41] Van Aart A.E., Velkers F.C., Fischer E., Broens E.M., Egberink H., Zhao S., Engelsma M., Hakze-van der Honing R.W., Harders F., de Rooij M. (2021). SARS-CoV-2 infection in cats and dogs in infected mink farms. Transbound. Emerg. Dis..

[42] Chaintoutis S.C., Thomou Z., Mouchtaropoulou E., Tsiolas G., Chassalevris T., Stylianaki I., Lagou M., Michailidou S., Moutou E., Koenen J. (2021). Outbreaks of SARS-CoV-2 in naturally infected mink farms: Impact, transmission dynamics, genetic patterns, and environmental contamination. PLoS Pathog..

[43] Larsen H.D., Fonager J., Lomholt F.K., Dalby T., Benedetti G., Kristensen B., Urth T.R., Rasmussen M., Lassaunière R., Rasmussen T.B. (2021). Preliminary report of an outbreak of SARS-CoV-2 in mink and mink farmers associated with community spread, Denmark, June to November 2020. Eurosurveillance.

[44] Domańska-Blicharz K., Orłowska A., Smreczak M., Niemczuk K., Iwan E., Bomba A., Lisowska A., Opolska J., Trębas P., Potyrało P. (2021). Mink SARS-CoV-2 Infection in Poland-Short Communication. J. Vet. Res..

[45] Delahay R.J., de la Fuente J., Smith G.C., Sharun K., Snary E.L., Flores Girón L., Nziza J., Fooks A.R., Brookes S.M., Lean F. (2021). Assessing the risks of SARS-CoV-2 in wildlife. One Health Outlook.

[46] De Rooij M., Hakze-Van der Honing R.W., Hulst M.M., Harders F., Engelsma M., van de Hoef W., Meliefste K., Nieuwenweg S., Oude Munnink B.B., van Schothorst I. (2021). Occupational and environmental exposure to SARS-CoV-2 in and around infected mink farms. Occup. Environ. Med..

[47] OIE SARS-CoV-2 in Animals Used for Fur Farming. https://www.oie.int/app/uploads/2021/03/glews-risk-assessment-fur-animals-SARS-CoV-2.pdf.

[48] Boklund A., Gortázar C., Pasquali P., Roberts H., Nielsen S.S., Stahl K., Stegeman A., Baldinelli F., Broglia A. (2021). Monitoring of SARS-CoV-2 infection in mustelids. EFSA J..

[49] Maran T., Skumatov D., Gomez A., Põdra M., Abramov A.V., Dinets V. (2016). Mustela lutreola. The IUCN Red List of Threatened Species. https://www.iucnredlist.org/species/14018/45199861.

[50] Zuberogoitia I., Zalewska H., Zabala J., Zalewski A. (2013). The impact of river fragmentation on the population persistence of native and alien mink: An ecological trap for the endangered European mink. Biodivers. Conserv..

